# Supplementary material for: Recent hemispheric asymmetry in global ocean warming induced by climate change and internal variability
Source: Nat Commun. 2020 Apr 24;11:2008. doi: 10.1038/s41467-020-15754-3 (PMC7181756; doi:10.1038/s41467-020-15754-3)
Supplement: Supplementary file 1 — Supplementary Information [file 41467_2020_15754_MOESM1_ESM.pdf]

Supplementary Information for

Recent hemispheric asymmetry in global ocean warming induced by climate change and internal variability

Rathore et al.

Supplementary Notes

Supplementary Note 1 Ocean heat content anomaly trend of 0-2000 m depth from the RCP 8.5 (2006-2015) simulations of 11 CMIP5 models used in this study

It is also interesting to look at the simulated OHC trend pattern for 0-2000 m using the RCP 8.5 scenario from 11 CMIP5 models (Supplementary Fig. S4). Each of the individual simulations has internal variability with varying phase and a slow climate change signal from anthropogenic forcing superimposed on it.

Most of the individual model simulations show a warmed North Atlantic and the signature of one phase or another of decadal variability from Pacific Decadal Oscillation/Inter-decadal Pacific Oscillation (PDO/IPO) in the tropical Pacific (Supplementary Fig. S4). In the different models, much of the internal variability comes from the signature of ENSO and IPO, and this variability is model-dependent. Numerical simulations show that these internal variations arise from instabilities in distinct components of the climate system (e.g., ocean or atmosphere) or from interactions between the different components (e.g., ENSO). These internal variations in the climate system play a key role in climate variability on hemispheric, regional and smaller scales[12–15].

Supplementary Note 2 An alternative approach for the detection and attribution

The anti-symmetric hemispheric mode of internal variability can be further exploited to separate the distribution of internal variations from the climate change mode (e.g., Fig. 3a). We are proposing an alternative method to understand these two components. In this method, we choose to rotate the coordinate axes of Fig. 3a. in the direction of climate change, i.e. along the best fit line for historical and RCP simulation as shown in Fig. 3a. The horizontal axis represents the direction of climate change and normal axis broadly represents the internal variability (vertical axis on Supplementary Fig. S7 a). This representation is not perfect since the the rotated cloud from the Pi-control is not perfectly aligned with the vertical axis.

Supplementary Fig. S7b represents Fig. 3b but in rotated space for 0-2000 m depth range. The observed trend (red circle) represents the present state of global ocean warming which lies well inside the cloud of climate change (orange cloud) but also has a substantial contribution from the internal variability (green cloud). The trajectory of the 10-year running trends of the OHC shows the transition from the internal variability driven to the anthropogenic forcing driven OHC changes (Supplementary Fig. S7b). From the perspective of climate change, the present state of the ocean warming is consistent with the best estimates of RCPs for the 2006-2015 period (square symbols) and has a significant negative projection on the internal variability mode. This can be interpreted as the inter-hemispheric asymmetry due to the reduced rate of ocean heat gain in the northern hemisphere and enhanced in the southern hemisphere.

For further investigation, we draw the probability distribution curves for internal variability and climate change due to external forcing (Supplementary Fig. S7 c-d). Note the larger 95% confidence interval in the direction of internal variability (vertical axis) as compared to the projection of internal variability on the climate change axis (horizontal axis). This demonstrates the increased detectability of the climate change signal in this rotated coordinate system. The distribution of internal variability is shown in (Supplementary Fig. S7c) with the projected component of internal variability in observed trend, historical (Hist-CMIP5), and RCP simulations. The magnitude of the historical and RCP simulation on the curve (Supplementary Fig. S7c) shows that it is very likely that the contribution from internal variability is quite small in this coordinate system. However, the component of internal variability in the observed global ocean heat gain for this period is anomalously large.

In contrast to internal variability (Supplementary Fig. S7c), the probability distribution in the climate change direction (Supplementary Fig. S7d) shows that the internal variability cannot explain the observed warming. However, if we add anthropogenic forcing to the probability distribution curve from the internal variability, this shifts the curve to the right on the climate change axis, as shown by

the orange cloud (Supplementary Fig. S7d). This result is in striking agreement between the observed OHC trend and the anthropogenic warming (and its distribution) in the RCP projections of the same decade. We conclude that the internal variability redistributes the global ocean heat within the coupled climate system while the extra heat gained by the global system is fully accounted by anthropogenic forcing alone.

Supplementary Table

Supplementary Table 1 The CMIP5 Models with Pre-industrial Control Simulation Length. CMIP5 models used in this study and the length (years) of their pre-industrial control simulations.

Model	Modelling Centre	Pre-industrial control Length (yr)
ACCESS 1-0[1]	CSIRO-BoM	500
bcc-csm1-1[2]	BCC	500
CCSM4[3]	NCAR	1051
CMCC-CM[4]	Centro Euro-Mediterraneo per I Cambiamenti Climatici	330
CNRM-CM5[5]	Centre National de Recherches Meteorologiques	850
CSIRO-Mk3-6-0[6]	CSIRO-QCCCE	500
EC-EARTH[7]	EC-EARTH	452
GFDL-ESM2G[8]	NOAA/GFDL	500
MPI-ESM-LR[9]	MPI for Meterology	1000
MRI-CGCM3[10]	Meteorological Research Institute (MRI)	500
NorESM1-M[11]	Norwegian Climate Centre (NCC)	501

Supplementary Figures

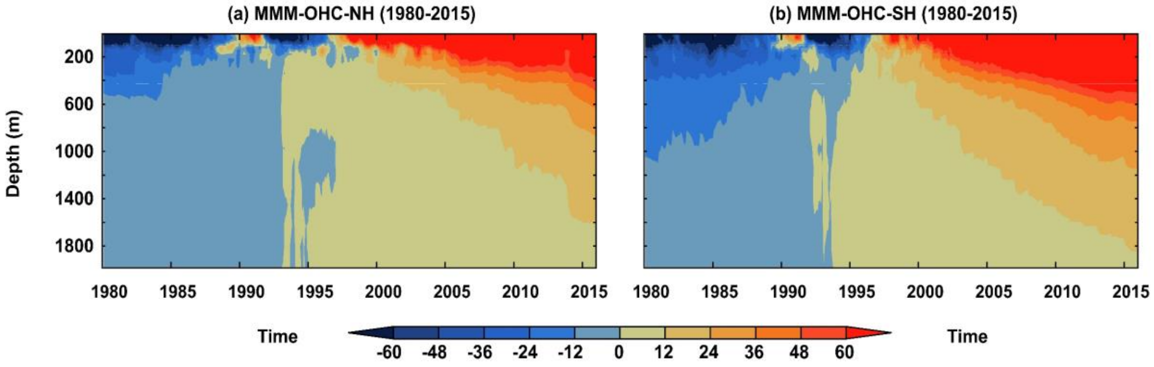

Supplementary Figure 1 Temporal variations of 0-2000 m ocean heat content anomaly. Hovmöller plot (depth vs time) of 0-2000 m ocean heat content anomaly (10^{18} J m^{-1}) during 1980-2015 and referenced from 1980-2005 for **(a)** northern hemisphere (NH) and **(b)** southern hemisphere (SH)

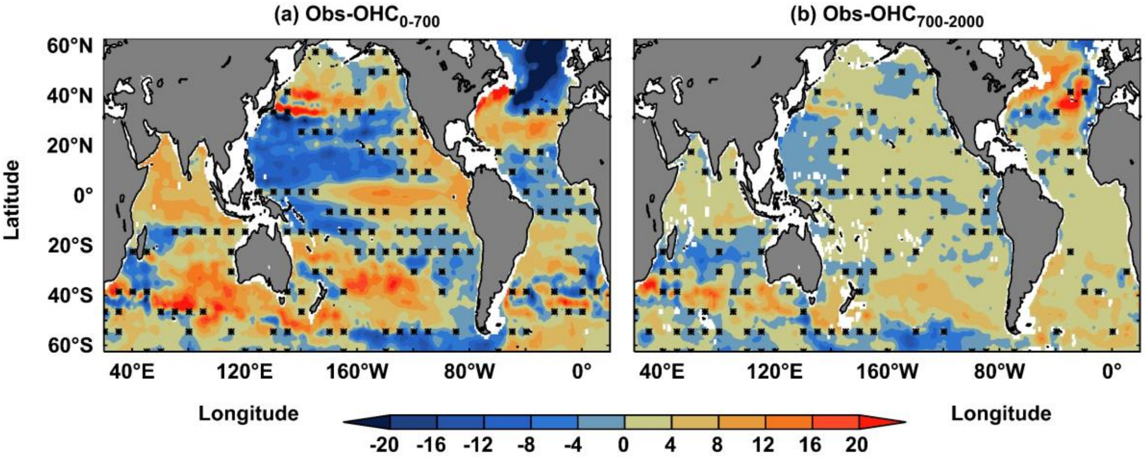

Supplementary Figure 2 Linear temporal trend in ocean heat content anomaly. Spatially observed linear trend of global ocean heat content anomaly (10^7 J m^{-2} $year^{-1}$) during 2005-2015 for **(a)** 0-700 m and **(b)** 700-2000 m. Stippling indicates the locations where OHC anomaly trends are not significant i.e. less than 2*standard error of the trends estimated from (n = 6) observation products used in this study.

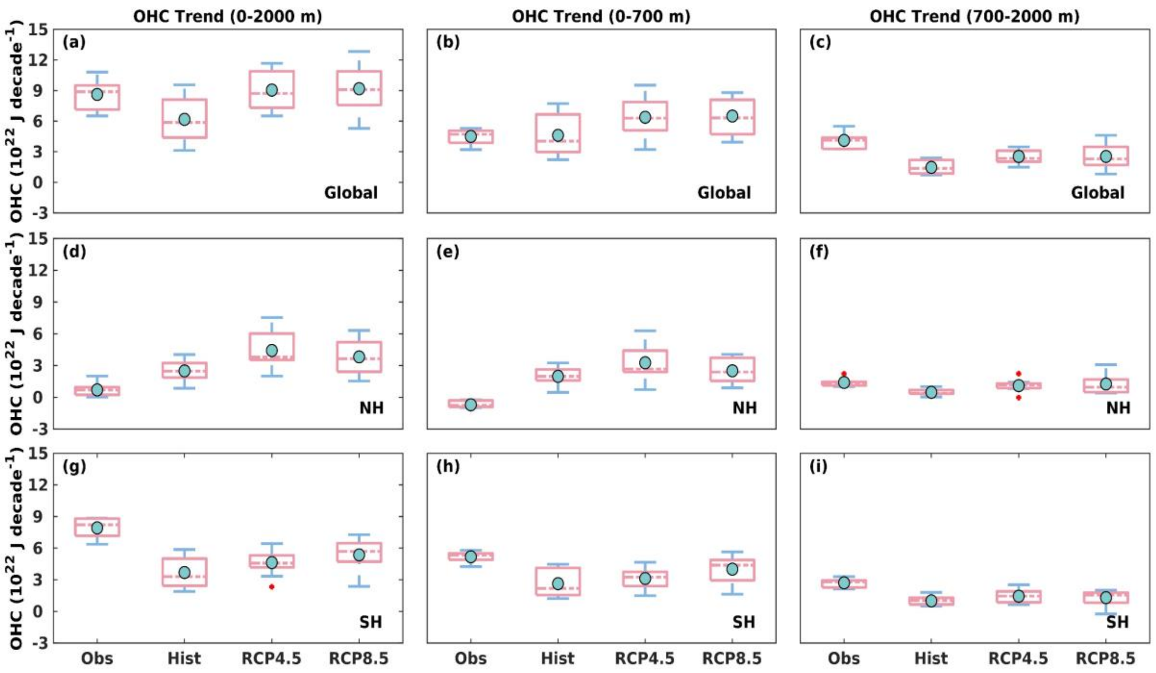

Supplementary Figure 3 Comparison of the trends of ocean heat content anomaly. Whisker plot of linear trend of the ocean heat content anomaly (10^{22} J decade^{-1}) in global, northern and southern hemisphere ocean for **(a, d, g)** 0-2000 m depth, **(b, e, h)** 0-700 m depth and **(c, f, i)** 700-2000 m depth. The red dots represent the extreme data point lying at the farthest distance from the median (dash line in boxes) and cyan color circles represents the ensemble mean trend from observations (2005-2015), historical (1980-2005) and RCPs (2006-2015) simulation. The horizontal lines of Whiskers

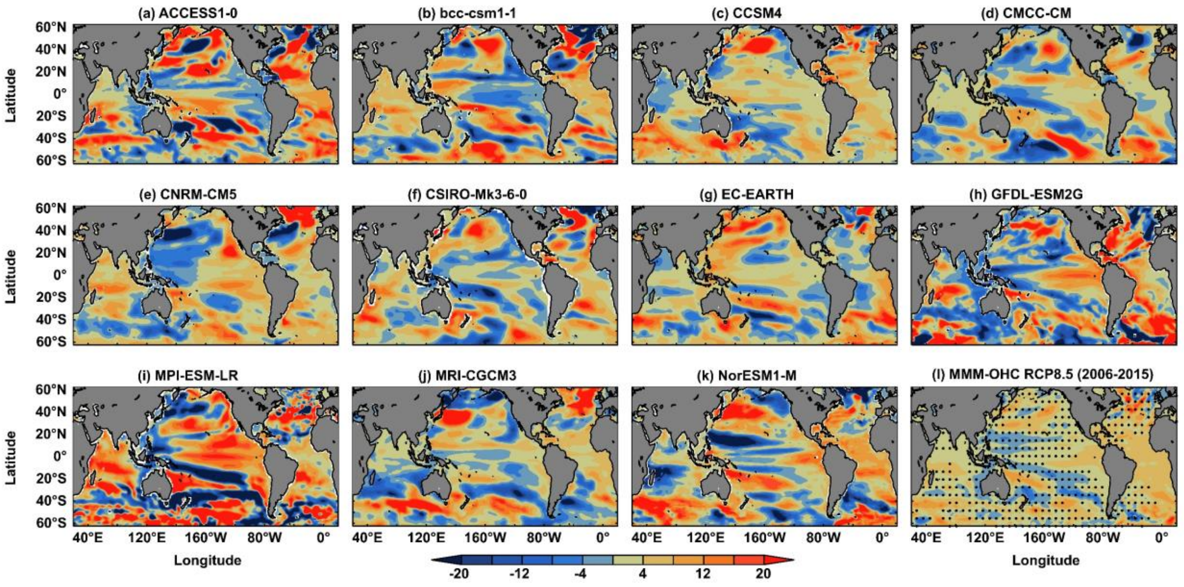

Supplementary Figure 4 Ocean heat content anomaly trend of 11 CMIP5 models. Linear trend of depth integrated ocean heat content (0-2000 m, 10^{7} J m^{-2} $year^{-1}$) from RCP 8.5 simulation (2006-2015) of 11 CMIP5 models used in this study. Panel **(l)** represents the multi model mean trend with the Stippling indicates the locations where OHC anomaly trends are not significant i.e. less than 2*standard error of the trends estimated from (n = 11) CMIP5 models used in this study.

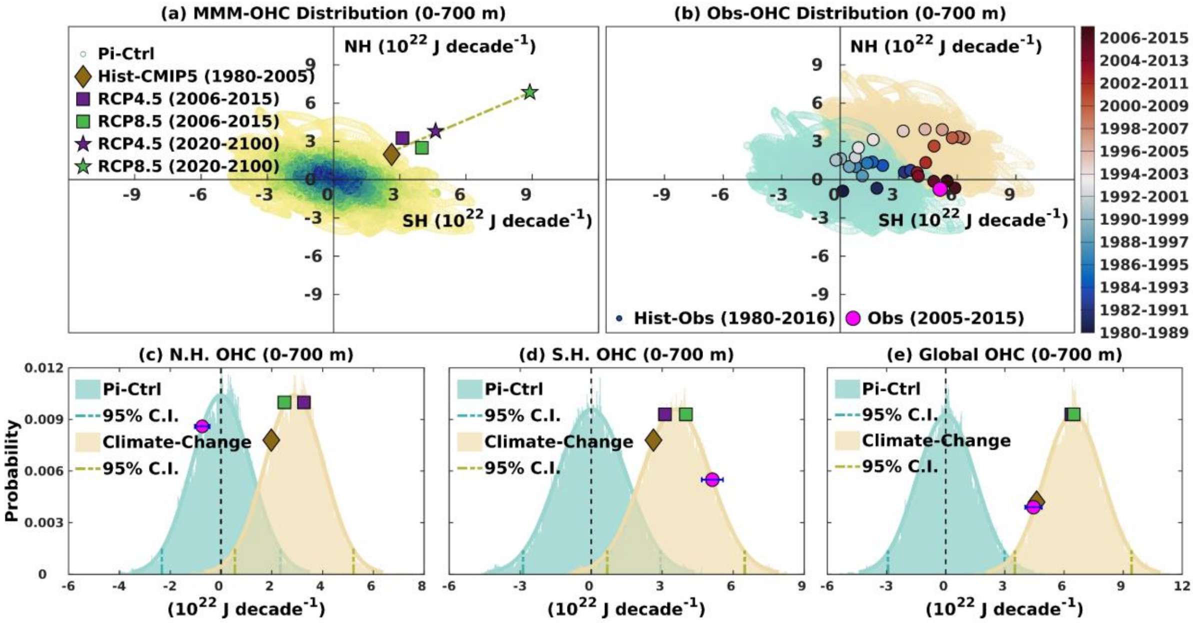

Supplementary Figure 5 Probabilistic analysis of the ocean heat content anomaly trend. Distribution of the linear trend of the OHC anomaly (10^{22} J decade^{-1}) for the depth of 0-700 m for Northern (NH) and Southern Hemisphere (SH) from **(a)** Multi model ensemble (MME) of pre-industrial control (Pi-Ctrl) simulation (cloud), with the Multi Model Mean (MMM) trend from the historical (Hist-CMIP5, brown diamond, 1980-2005), RCP 4.5 (purple square 2006-2015; purple star 2020-2100) and RCP 8.5 (green square 2006-2015; green star 2020-2100) simulation, and the least square fit line passing through these points to represent the direction of climate change **(b)** the green cloud is the same as shown in (a) and the orange cloud is the represent the climate change signal in the direction of the best fit line as shown in (a), Observed trend over the period of 2005-2015 (Obs, pink circle) along with the trajectory (scatter dots) of the 10 year running trends from the long-term observations over the period of 1980-2016 (Hist-Obs). **(c)** Probability distribution curve for the northern hemisphere's internal variability (green cloud in panel b) and climate change (orange cloud in panel b) with the OHC trend from observations (pink circle with the error bar of 95% confidence intervals, 2005-2015), MMM of historical (brown diamond, 1980-2005), RCP 4.5 (purple square, 2006-2015) and RCP 8.5 (green square, 2006-2015) simulations. **(d)** and **(e)** Same as (c) but for the Southern Hemisphere and the Global Ocean respectively. The 95% confidence interval for the probability distribution curves is derived from the 2-sigma limits for the gaussian distribution of OHC trend.

193

194

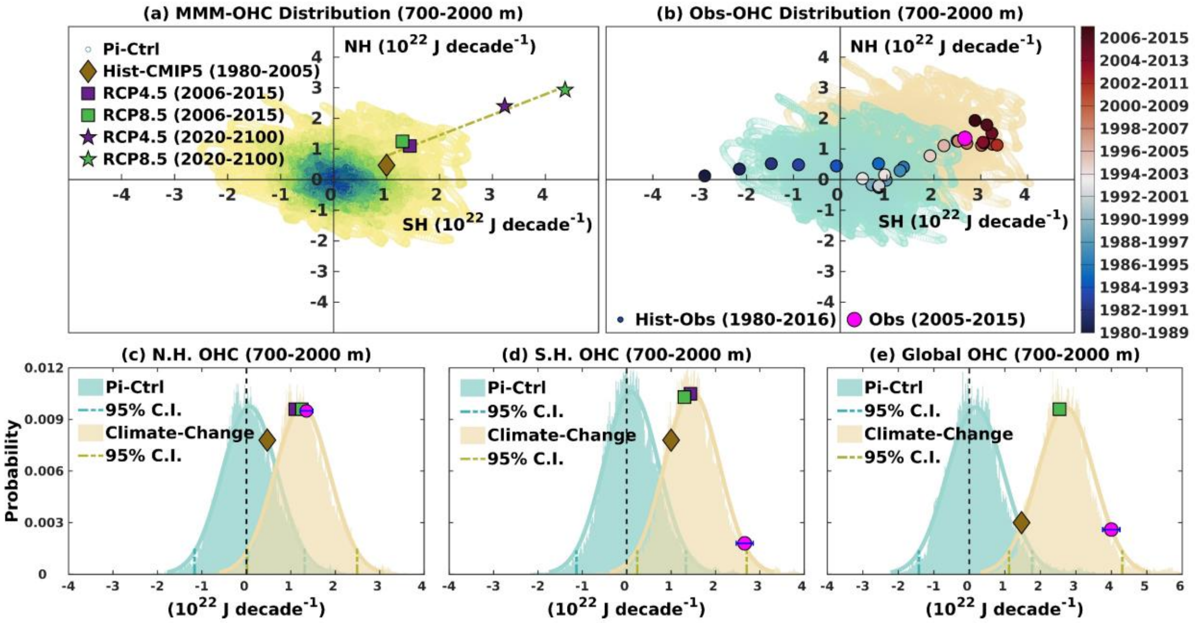

Supplementary Figure 6 Probabilistic analysis of the ocean heat content anomaly trend. Distribution of the linear trend of the OHC anomaly (10^{22} J decade^{-1}) for the depth of 700-2000 m for Northern (NH) and Southern Hemisphere (SH) from **(a)** Multi model ensemble (MME) of pre-industrial control (Pi-Ctrl) simulation (cloud), with the Multi Model Mean (MMM) trend from the historical (Hist-CMIP5, brown diamond, 1980-2005), RCP 4.5 (purple square 2006-2015; purple star 2020-2100) and RCP 8.5 (green square 2006-2015; green star 2020-2100) simulation, and the least square fit line passing through these points to represent the direction of climate change **(b)** the green cloud is the same as shown in (a) and the orange cloud is the represent the climate change signal in the direction of the best fit line as shown in (a), Observed trend over the period of 2005-2015 (Obs, pink circle) along with the trajectory (scatter dots) of the 10 year running trends from the long-term observations over the period of 1980-2016 (Hist-Obs). **(c)** Probability distribution curve for the northern hemisphere's internal variability (green cloud in panel b) and climate change (orange cloud in panel b) with the OHC trend from observations (pink circle with the error bar of 95% confidence intervals, 2005-2015), MMM of historical (brown diamond, 1980-2005), RCP 4.5 (purple square, 2006-2015) and RCP 8.5 (green square, 2006-2015) simulations. **(d)** and **(e)** Same as (c) but for the Southern Hemisphere and the Global Ocean respectively. The 95% confidence interval for the probability distribution curves is derived from the 2-sigma limits for the gaussian distribution of OHC trend.

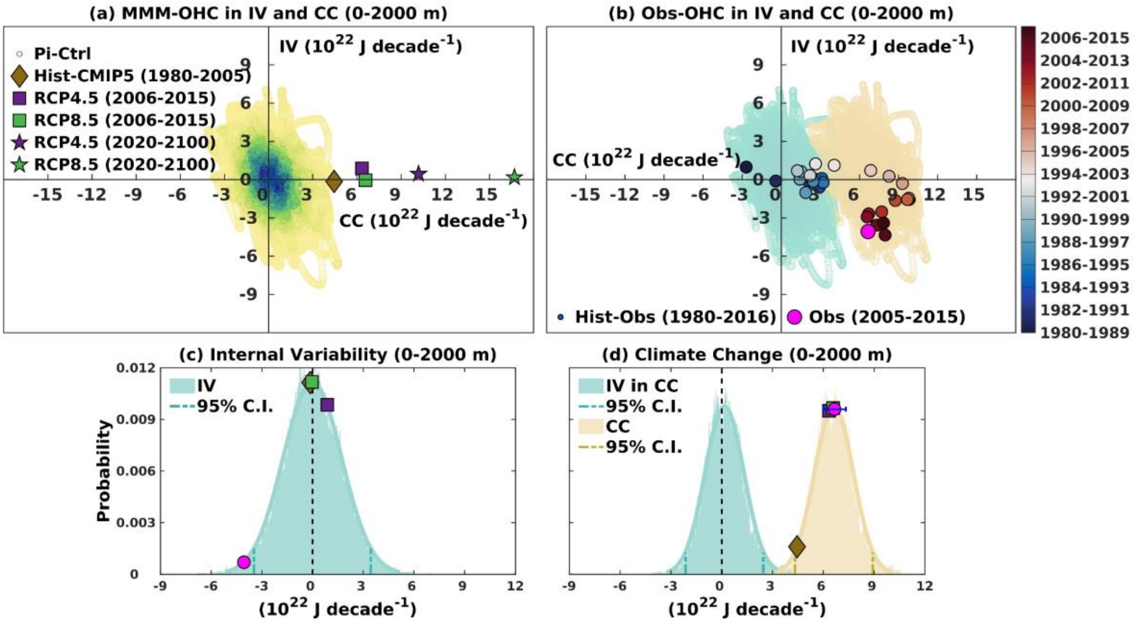

Supplementary Figure 7 Alternative approach for detection and attribution. (a) Rotation of (Fig. 3a) in the direction of least square fit from the Historical and RCP simulation to get the Climate Change (CC) and Internal Variability (IV) axis with all the points in rotated space **(b)** same as (Fig. 3b) but in rotated space defined by the climate change and internal variability axes **(c)** Probability distribution curve along the Internal Variability axis and all the points denotes the amount of Internal Variability with its likelihood **(d)** same as (c) but for the Internal Variability along the Climate Change axis (green cloud) and its translation (orange cloud) by adding the average of the multi model mean trend of RCP 4.5 and RCP 8.5 for the period of 2006-2015 $\left(\frac{RCP4.5_{MMM}+RCP8.5_{MMM}}{2}\right)_{2006\text{-}2015}$ to represent the climate change signal. The 95% confidence interval for the probability distribution curves is derived from the 2-sigma limits for the gaussian distribution of OHC trend derived from random selection of 10-year blocks.

Supplementary References

1. Bi, D. *et al.* The ACCESS coupled model: description, control climate and evaluation. *Aust. Meteorol. Oceanogr. J.* **63**, 41–64 (2013).

2. Xiao-Ge, X. *et al.* How Well does BCC_CSM1.1 Reproduce the 20th Century Climate Change over China? *Atmos. Ocean. Sci. Lett.* **6**, 21–26 (2013).

3. Gent, P. R. *et al.* The community climate system model version 4. *J. Clim.* **24**, 4973–4991 (2011).

4. Scoccimarro, E. *et al.* Effects of tropical cyclones on ocean heat transport in a high-resolution coupled general circulation model. *J. Clim.* **24**, 4368–4384 (2011).

5. Voldoire, A. *et al.* The CNRM-CM5.1 global climate model: Description and basic evaluation. *Clim. Dyn.* **40**, 2091–2121 (2013).

6. Jeffrey, S. *et al.* Australia's CMIP5 submission using the CSIRO-Mk3.6 model. *Aust. Meteorol. Oceanogr. J.* **63**, 1–13 (2013).

7. Prodhomme, C. *et al.* Benefits of increasing the model resolution for the seasonal forecast quality in EC-earth. *J. Clim.* **29**, 9141–9162 (2016).

8. Dunne, J. P. *et al.* GFDL's ESM2 global coupled climate-carbon earth system models. Part I: Physical formulation and baseline simulation characteristics. *J. Clim.* **25**, 6646–6665 (2012).

9. Jungclaus, J. H. *et al.* Characteristics of the ocean simulations in the Max Planck Institute Ocean Model (MPIOM) the ocean component of the MPI-Earth system model. *J. Adv. Model. Earth Syst.* **5**, 422–446 (2013).

10. Yukimoto, S. *et al.* A new global climate model of the Meteorological Research Institute: MRI-CGCM3: -Model description and basic performance-. *J. Meteorol. Soc. Japan* **90**, 23–64 (2012).

11. Bentsen, M. *et al.* The Norwegian Earth System Model, NorESM1-M – Part 1: Description and basic evaluation of the physical climate. *Geosci. Model Dev.* **6**, 687–720 (2013).

12. Palmer, M. D. & McNeall, D. J. Internal variability of Earth's energy budget simulated by CMIP5 climate models. *Environ. Res. Lett.* **9**, (2014).

13. Bindoff, N. L. *et al.* Detection and attribution of climate change: From global to regional. in *Climate Change 2013 the Physical Science Basis: Working Group I Contribution to the Fifth Assessment Report of the Intergovernmental Panel on Climate Change* **9781107057**, 867–952 (Cambridge University Press, Cambridge, United Kingdom and New York, NY, USA, 2013).

14. Lyu, K., Zhang, X., Church, J. A. & Hu, J. Quantifying internally generated and externally forced climate signals at regional scales in CMIP5 models. *Geophys. Res. Lett.* **42**, 9394–9403 (2015).

15. Sutton, R., Suckling, E. & Hawkins, E. What does global mean temperature tell us about local climate? *Philos. Trans. R. Soc. A Math. Phys. Eng. Sci.* **373**, (2015).
